# Adaptation and Resistance: How *Bacteroides thetaiotaomicron* Copes with the Bisphenol A Substitute Bisphenol F

**DOI:** 10.3390/microorganisms10081610

**Published:** 2022-08-09

**Authors:** Sarah Riesbeck, Hannes Petruschke, Ulrike Rolle-Kampczyk, Christian Schori, Christian H. Ahrens, Christian Eberlein, Hermann J. Heipieper, Martin von Bergen, Nico Jehmlich

**Affiliations:** 1 Helmholtz-Centre for Environmental Research—UFZ GmbH, Department of Molecular Systems Biology, 04318 Leipzig, Germany; 2 Agroscope, Molecular Ecology and SIB Swiss Institute of Bioinformatics, 8820 Wädenswil, Switzerland; 3 Proteomics Core Facility, Biozentrum, University of Basel, 4056 Basel, Switzerland; 4 Helmholtz-Centre for Environmental Research—UFZ GmbH, Department of Environmental Biotechnology, 04318 Leipzig, Germany; 5 Institute of Biochemistry, Faculty of Biosciences, Pharmacy and Psychology, University of Leipzig, 04103 Leipzig, Germany

**Keywords:** xenobiotics, bisphenols, gut microbiome, fatty acid methyl ester, short-chain fatty acids, proteomics

## Abstract

Bisphenols are used in the process of polymerization of polycarbonate plastics and epoxy resins. Bisphenols can easily migrate out of plastic products and enter the gastrointestinal system. By increasing colonic inflammation in mice, disrupting the intestinal bacterial community structure and altering the microbial membrane transport system in zebrafish, bisphenols seem to interfere with the gut microbiome. The highly abundant human commensal bacterium *Bacteroides thetaiotaomicron* was exposed to bisphenols (Bisphenol A (BPA), Bisphenol F (BPF), Bisphenol S (BPS)), to examine the mode of action, in particular of BPF. All chemicals caused a concentration-dependent growth inhibition and the half-maximal effective concentration (EC50) corresponded to their individual logP values, a measure of their hydrophobicity. *B. thetaiotaomicron* exposed to BPF decreased membrane fluidity with increasing BPF concentrations. Physiological changes including an increase of acetate concentrations were observed. On the proteome level, a higher abundance of several ATP synthase subunits and multidrug efflux pumps suggested an increased energy demand for adaptive mechanisms after BPF exposure. Defense mechanisms were also implicated by a pathway analysis that identified a higher abundance of members of resistance pathways/strategies to cope with xenobiotics (i.e., antibiotics). Here, we present further insights into the mode of action of bisphenols in a human commensal gut bacterium regarding growth inhibition, and the physiological and functional state of the cell. These results, combined with microbiota-directed effects, could lead to a better understanding of host health disturbances and disease development based on xenobiotic uptake.

## 1. Introduction

Bisphenols are used in the process of polymerization of polycarbonate plastics and epoxy resins. The annual production volume of bisphenols is one of the highest worldwide, with a production of bisphenol A of approximately 1.15 million tons (BPA; 4,4′-isopropylidenediphenol) in Europe alone in 2015 [1]. BPA has been classified as an endocrine-disrupting chemical (EDC), associated with cardiovascular disease [2], metabolic syndrome, and obesity [3,4]. It has been banned from products for infants and food contact materials due to growing concern in several countries [5], which lead to the intensified use of structurally similar substitutes. Particularly, bisphenol S (BPS; 4,4′-sulfonyldiphenol) replaced BPA as a developer in thermal paper [6]. Bisphenol F (BPF; bis(4-hydroxyphenyl)methane) is widely used in lacquers, varnishes, water pipes, dental sealants, and food packaging [7]. Bisphenols are frequently detected in the environment, occurring in surface water, sediments, and wastewater, with higher concentrations in urban industrial areas [8]. Lee et al. [9] identified BPF as the most abundant bisphenol in domestic wastewater treatment plants in Korea with concentrations up to 1780 ng g^−1^ dry weight. Further, concentrations of BPF found in surface water and seawater samples in Japan (up to 2850 ng/L), Korea (up to 1300 ng/L), and China (up to 1110 ng/L), exceeded the concentrations of BPA found in all the sampling sites of each country by far [10], likely indicating that BPF is the main substitute for BPA in South-East Asian regions.

The migration of BPA, BPS, and BPF into food has been described (e.g., dairy products, meat, fish, vegetables, canned food) [11,12,13]. Dietary intake has been presumed the primary exposure route for humans, with estimated daily intakes (EDI) decreasing with age [7]. Based on urinary concentrations, the EDI solely from diet for BPA was calculated for infants to range from 0.01 to 13 µg/kg/d and for adults from 0.1 to 4.2 µg/kg/d [7]. Modelling the daily intake for pharmacokinetic properties for BPS, Oh et al. [14] assumed similar characteristics as for BPA, but with a longer retention time in the human body for BPS. Both BPS and BPF have been detected in comparable urinary concentrations to BPA [15,16]. The temporary tolerable daily intake (t-TDI) of BPF is orientated at the TDI of BPA (4 µg/kg body weight/day), whereas the TDI of BPS is set at 50 µg/kg body weight/day, due to its lesser hormonal potencies.

Once they have entered the human body, BPA and BPS are excreted with a half-life of urinary elimination of 5.4 h and 7 h, respectively [14,17,18]. It should be noted that this varies with age and sex [19,20]. However, BPA has been found in brain, liver, adipose tissue [21,22], and body fluids [23], illustrating its environmental prevalence. BPF has been reported to migrate into the reproductive tracts in rats [24]. BPF has shown similar estrogenic activities as BPA. After oral administration in rats, it alters thyroid hormone levels [25]. BPF has been associated with immune system toxicity [26], but the majority of studies still focus on BPS and BPA. Considering a similar or even stronger estrogenic potency of BPF compared to BPA, it is a concerning chemical, which should be considered for further toxicological studies [27,28].

In adult zebrafish, BPF and BPS accumulated with the highest levels found in the intestine [29]. Here, xenobiotics can affect the gut microbiome and/or get modified by the diverse bacterial enzymatic capacities [30]. Being involved in crucial roles of immunity [31] and homeostatic host physiology and metabolism [32], the gut microbiome is a key organ in human health. Changes in the bacteria–human cross-talk by altered production of microbial metabolites and peptides are linked with several human diseases [33]. Administering 50 µg/kg BPA to mice reduced microbial metabolites derived from aromatic acids in the colon and increased colonic inflammation [34].

In zebrafish larvae, BPF and BPS showed a concentration-dependent disruption of the intestinal community structure and alterations in the microbial membrane transport system, and xenobiotic degradation and metabolism. In particular, an increase in ATP-binding cassette (ABC) transporters and bacterial secretion systems was observed [35]. Environmentally relevant concentrations of BPS showed an effect at the proteome level, regarding ABC transporters involved in the transport of cell wall components [36].

For xenobiotics, the first barrier to overcome is the cell membrane, damage leading to altered cell function or ultimately, cell death. Hereby, the hydrophobicity of a compound, given as its logP value, is directly correlated to its accumulation in the phospholipid bilayer and consequently to cell toxicity [37]. BPA was shown to aggregate into clusters, pulling phospholipids from bilayer membranes and eventually leading to pore formation [38]. Regarding similar chemical properties of BPF and BPA (including e.g., logP: 2.91 and 3.40, respectively), they are expected to exhibit a similar permeation through biological membranes [39,40].

The human commensal bacterium *Bacteroides thetaiotaomicron* is a Gram-negative obligate anaerobe, inhabiting the outer mucus layer of the human gut. It is a member of one of the most prominent phyla (*Bacteroidetes*) in the human gut that, together with the phylum of Firmicutes, makes up 90% of the bacteriome [41]. Intestinal mucus is a defensive barrier between gut epithelial and bacteria comprised of glycoproteins. Recently, it has been shown that its maturation and function are modulated by the gut microbiota associated with it [42,43]. *B. thetaiotaomicron* ferments complex carbohydrates (i.e., starch, dietary fiber) into short-chain fatty acids (SCFAs), known immunomodulatory and signaling metabolites [44]. Providing also for microbes located close to them, *Bacteroidetes* are a major player in the food network inside the gut, and a decrease is associated with obesity [45].

Due to the increasing production volume of BPA substitutes in recent years, these substitutes have become more abundant in the environment and in organisms. Studies regarding the effects of BPA analogues on the gut microbiome are rare, as most studies investigate the impact of BPA. Showing similar lipophilicity and endocrine-disrupting effects as BPA, BPF is a relevant target for further toxicological studies. Disturbance of the gut microbiota in terms of community structure and function is linked to several diseases, and the impact of xenobiotics on gut bacteria should be considered for risk assessment.

In this study, we exposed *B. tethaiotaomicron* to BPA, BPF, and BPS. First, we compared the bisphenols based on growth inhibition in adapted concentration series deduced by their logP values. Second, we focused on the effects of BPF exposure, with respect to (1) a change in membrane lipid composition, (2) a shift in short-chain fatty acid production, and (3) alterations in the functional profile based on protein abundances and pathway analyses.

## 2. Materials and Methods

### 2.1. Bacterial Strain and Growth Condition

*Bacteroides thetaiotaomicron* (DSM 2079) was grown in Brain–Heart-Infusion (BHI) under anaerobic conditions as described in [36] (Supplementary Materials Table S1).

### 2.2. Bisphenol Exposure and Sampling

For microbial growth, bisphenols were added in different concentrations to anaerobic cultivation tubes. For lipid analysis, Bisphenol A (Sigma Aldrich, St. Louis, MO, USA) was added from 0.05 to 0.6 mM in 0.05 intervals. Bisphenol F (Sigma Aldrich) was added in following range: 0.125, 0.25, 0.375, 0.5, 0.75, 1, 1.25, 1.5, 1.75, 2 mM. Bisphenol S (Sigma Aldrich, St. Louis, MO, USA) was added as follows: 0.5, 1, 1.5, 2, 2.5, 3, 3.5, 4, 4.5, 5, 6 mM. All bisphenols were dissolved in ethanol and control tubes were supplemented with 0.5% ethanol (*v*/*v*) equivalent to chemical exposure tubes. For proteomics analysis, tubes were supplied with 0.57 mM BPF corresponding to 200 µg/kg body weight. Two concentrations of BPF, 0.14 and 0.57 mM, were used for Short-Chain Fatty Acid analysis, corresponding to 50 and 200 µg/kg body weight, respectively. For chemical uptake, we calculated with an average person with 70 kg body weight. We assumed the total amount of BPF reaches the gut and accumulates in the stool. Hence, we considered the average amount of stool per day [46] and final concentrations were adjusted to the culture medium (calculations see Supplementary Table S2).

For all approaches, *B. thetaiotaomicron* was inoculated to an initial OD_600nm_ of ~0.1. The bacterial growth was monitored (see Supplementary Material Table S3) and cells were harvested in the mid-to-late exponential phase. For lipid analysis, three or four individual cultures of the same concentration were pooled to obtain the required cell number of 10^10^–10^11^ cells. Cultures for other analysis were sampled individually. Samples were centrifuged at 5000× *g* at 4 °C for 10 min. Pellets for lipid analysis were washed with cold 1× PBS and centrifuged again. Pellets and supernatant were immediately frozen at −20 °C or −80 °C, respectively, until sample preparation.

### 2.3. Growth Rates

The growth rate constant (µ) for each bisphenol and concentration was calculated using the OD_600_ values of the endpoint of logarithmic growth (*N_t_*), of the first measuring point (*N*_0_) and the referring time points in hours (*t*, *t*_0_).

$$µ=\frac{\ln\left(N_{t}\right)-\ln\left(N_{0}\right)}{\left(t-t_{0}\right)}$$

The effect of bisphenols on overall growth was determined by differences in the growth rates of cultures treated with bisphenols (µ_1_) and with growth controls (µ_0_) treated with 0.5% (*v*/*v*) ethanol. The growth rate was determined as the percentage of growth rates of bacteria in the presence of bisphenols and the growth control cultures.

$$Relative\;growth\;rate\;\left(\%\right):\;\frac{µ1,bisphenols}{µ0,\;control}*100$$

### 2.4. Lipid Analysis

#### 2.4.1. Lipid Extraction and Derivatization to Fatty Acid Methyl Esters (FAME)

The extraction of membrane lipids and derivatization of fatty acids was carried out according to the method of Bligh and Dyer [47]. Thirty to 40 mL of bacterial culture were pelleted 5000× *g* at 4 °C for 10 min, washed with cold 1× PBS, and centrifuged again. Lipids were extracted using chloroform/methanol/water as described in [47]. Fatty acids were then esterified with methanol by incubation at 95 °C for 15 min in boron trifluoride/methanol according to the method of Morrison and Smith [48]. Methylated fatty acids were extracted in hexane.

#### 2.4.2. Analysis of Fatty Acid Composition by GC-FID

Analysis was performed using a quadruple GC System (HP5890, Hewlett & Packard, Palo Alto, Santa Clara, CA, USA) equipped with a split/splitless injector. A CPSil 88 capillary column (Chrompack, Middelburg, The Netherlands; length, 50 m; inner diameter, 0.25 mm; 0.25 lm film) was used for the separation of the FAME. The GC injector temperature was held at 240 °C, detector temperature at 270 °C. The injection was splitless, carrier gas was helium at a flow of 2 mL/min. The temperature program was: 40 °C, 2 min isothermal; 8 °C/min to 220 °C; 15 min isothermal at 220 °C. The pressure program was: 27.7 psi (=186.15 kPa), 2 min isobaric; 0.82 psi/min (5.65 kPa/min) to the final pressure 45.7 psi; 15.55 min isobaric at 45.7 psi (310.26 kPa). The relative amount of FAMEs were calculated based on peak areas of the total ion chromatograms (TIC). For identification of fatty acids, authentic reference compounds obtained from Supelco (Bellefonte, PA, USA) were co-injected and identified by GC.

#### 2.4.3. Data Analysis

Relative abundance of saturated fatty acids was calculated by the ratio of saturated fatty acids (C14:0, C16:0 and C18:0) and unsaturated fatty acids (16:1cis, 18:1cis 18:1cis). Change in membrane saturation was also indicated by the ratio of saturated/branched fatty acids (SFA/BFA). The sum of the saturated fatty acids (C14:0, C16:0 and C18:0) divided to the sum of the branched fatty acids (C15:0iso and C15:0anteiso).

All visualizations were performed in RStudio (Version 4.0.2, Vienna, Austria) using ggplot. Relative growth inhibition µ_rel_ [%] was determined by comparing growth rates of cultures grown with bisphenols and control cultures supplied with 0.5% (*v*/*v*) ethanol. Growth rate was fitted by applying a linear regression model using RStudio. Diagnostic plots and linear equations are provided (Supplementary Figure S1).

### 2.5. Short-Chain Fatty Acid Analysis

#### 2.5.1. Metabolite Extraction

Short Chain Fatty Acids (SCFA) were measured as previously described [36]. Briefly, the samples were mixed with acetonitrile to a final concentration of 50% acetonitrile. SCFAs were derivatized with 0.5 volumes of 200 mM 3-nitrophenylhydrazine and 0.5 volumes of 120 mM N-(3-dimethylaminopropyl)-N′-ethylcarbodiimide hydrochloride in pyridine at 40 °C and 300 rpm for 30 min. The derivatized SCFA solutions were diluted 1:50 in 10% acetonitrile and measured.

#### 2.5.2. LC MS/MS Measurement and Data Analysis

In total, 10 µL of the diluted SCFA derivatives was injected into a RSLC UltiMate 3000^®^ system (ThermoFisher, Waltham, MA, USA) coupled on-line with a QTRAP 5500^®^ mass spectrometer (AB Sciex, Framingham, MA, USA). Chromatographic separation of SCFAs was performed on an Acquity UPLC BEH C18 column (1.7 µm; Waters, Eschborn, Germany) using A: H_2_O (0.01% formic acid, FA) and B: acetonitrile (0.01% FA) as the mobile phases. The flow rate was set to 0.35 mL/min, the column temperature held at 40 °C. The gradient elution was performed as follows: 2 min at 15% B, 15–50% B in 15 min, then held at 100% B for 1 min. Finally, the column was equilibrated for 3 min at 15% B. For identification and quantitation, a scheduled MRM method was used, with specific transitions for every SCFA. Peak areas were determined in Analyst^®^ Software (v1.6.2, AB Sciex) and areas for single SCFAs were exported. Metabolite quantification with calibration curves, normalization to OD_600_ and statistical analysis using an ANOVA followed by a Tukey’s HSD were performed with in-house written *R* scripts in RStudio (Version 4.0.2).

### 2.6. Proteomics

#### 2.6.1. Protein Extraction and Proteolytic Cleavage

Ten mL B. thetaiotaomicron culture was centrifuged (5000× *g*, 4 °C, 10 min) and the pellet was frozen at −20 °C. After three freeze thaw cycles in liquid nitrogen, the pellet was solved in 1 mL lysis buffer (8M Urea, 1 mM Phenylmethylsulfonylfluorid). Bacterial envelopes were disrupted by bead beating (FastPrep-24, MP Biomedicals, Sanra Ana, CA, USA; 5.5 ms, 1 min, 3 cycles) followed by ultra-sonication (UP50H, Hielscher, Teltow, Germany; cycle 0.5, amplitude 60%) and centrifugation (10,000× *g*, 10 min). The supernatant was used for protein concentration determination using the Pierce^TM^ 660 nm Protein Assay (Thermo Scientific, Thermo Fischer Scientific, Waltham, MA, USA).

Vivacon 500 columns (10 kDA MWCO membrane, Sartorius, Göttingen, Germany) were equilibrated with lysis buffer and centrifuged (14,000× *g*, 20 °C, 20 min). All following centrifugation steps were performed applying the same conditions. Sixty micrograms of protein were loaded onto the column and centrifuged. Proteins adherent to the filter were incubated with 200 µL 10 mM DTT in lysis buffer in a thermoshaker for 1 min with 600 rpm and for 30 min without shaking at 37 °C. After centrifugation, proteins were further alkylated and proteolytically cleaved as described in [49]. Peptides were solved in 0.1% formic acid for mass spectrometric measurement.

#### 2.6.2. Nano LC MS/MS Analysis

For each LC-MS/MS run, 5 μL of total peptide lysate was injected into nanoHPLC (UltiMate 3000 RSLCnano, Dionex, Thermo Fisher Scientific). Peptides were trapped on a C18-reverse phase trapping column (C18 PepMap100, 300 μm × 5 mm, particle size 3 μm (Thermo Fisher Scientific), followed by separation on a C18-reverse phase analytical column (Acclaim PepMap^®^ 100, 75 μm × 25 cm, particle size 3 μm, nanoViper (Thermo Fisher Scientific). Mass spectrometric analyses of eluted peptide lysates were performed on a Q Exactive HF mass spectrometer (Thermo Fisher Scientific) coupled with a TriVersa NanoMate (Advion, Harlow, UK) source in LC chip coupling mode as described [50].

#### 2.6.3. Data Analysis

Mass spectrometric data processing was performed using Proteome Discoverer (v.2.5, Thermo Fischer Scientific, USA) with SequestHT search engine. Search settings were set to trypsin (full), max. missed cleavage sites 2, precursor mass tolerance 10 ppm, and fragment mass tolerance 0.05 Da. Carbamidomethylation of cysteines was specified as a fixed modification. False discovery rates (FDR) were determined using Percolator [51]. Proteins were considered as identified when at least one unique peptide was found, the overall protein FDR was ≤0.01, and a SequestHT Score of ≥ 2 was reached. The protein coding sequences from *Bacteroides thetaiotaomicron* DSM 2079 (BioprojectX Accession: PRJNA543750) were downloaded from NCBI (https://www.ncbi.nlm.nih.gov, accessed on 8 February 2021) and accessions were converted to UniProt identifiers (http://www.uniprot.org/, accessed on 24 November 2021) and used as search database.

All visualizations and statistical analyses were performed in RStudio (Version 4.0.2) using in-house written scripts. Principal component analysis was performed using “prcomp” function with default setting and visualized by ggplot. Protein abundances were log2 transformed and median normalized (Supplementary Materials Figure S2). Proteins were detected as differentially expressed using unpaired t tests with a Benjamini-Hochberg adjusted *p*-value < 0.05 and a log2 fold change > 1. A result table of significantly differentially abundant proteins is provided (Supplementary Materials Table S4). Unique proteins found in control or treatment can be found in a supplementary list (Supplementary Materials Table S5). The pathway list of *Bacteroides thetaiotaomicron* was downloaded from Kyoto Encyclopedia of Genes and Genomes (KEGG) using the R package “KEGGREST”. Uniprot accessions from proteins were matched to KEGG pathways to identify functions of expressed proteins. Pathways with sufficient coverage (≥10%) on total per sample were considered for analysis. Statistical analysis was performed by using multiple unpaired *t* tests with a Benjamini–Hochberg adjusted *p*-value < 0.05, and log2 fold changes are provided (Supplementary Materials Table S6).

## 3. Results

### 3.1. Rate of Bacterial Growth Inhibition Depends on Hydrophobicity of Compound

**Figure 1:**
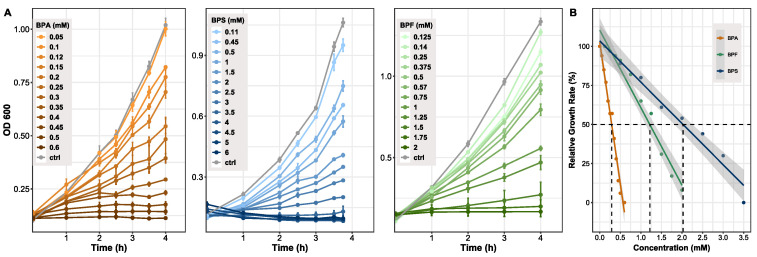
Growth of *Bacteroides thetaiotaomicron* exposed to BPA, BPF, and BPS. (**A**) Growth curves resulting from cells treated with different concentration of bisphenols. OD was measured at 600 nm and cells harvested in late exponential phase (*n* = 3). (**B**) Growth inhibition shown by relative growth rate (µ_rel_). Growth at 0 mM (control) was set to 100%. A linear fit was applied to determine the half maximal effective concentration (EC50) for each bisphenol. Grey area indicating 95% confidence interval. Adj. R^2^ = 0.97. EC50 values: BPA: 0.3 mM, BPF: 1.2 mM, BPS: 2 mM.

Determining differences between bisphenols in regard to overall growth inhibition of bacteria, *B. thetaiotaomicron* (*n* = 3) was exposed to several concentrations of bisphenols. Concentration ranges differed between BPA (0–0.6 mM), BPS (0–6 mM), and BPF (0–2 mM). All chemicals showed a concentration-dependent growth inhibition, becoming clearly visible in the late exponential phase of growth (Figure 1A). Based on the comparison of cultures grown with bisphenols and control cultures, the relative growth rates were determined (Figure 1B). Growth of control cultures corresponds to 100%. As a comparable measure of toxicity between compounds, the half maximal effective concentration (EC50) was evaluated, which clearly differed between the bisphenols. BPA showed the lowest EC50 value at 0.3 mM, BPF followed with a concentration of 1.2 mM, and BPS treated cells reached 50% of growth inhibition at 2 mM. Spectrophotometrically ascertained OD600 values and growth rates are provided (Supplementary Materials Table S3).

### 3.2. Membrane Adapts towards Rigidity Following BPF Exposure

**Figure 2:**
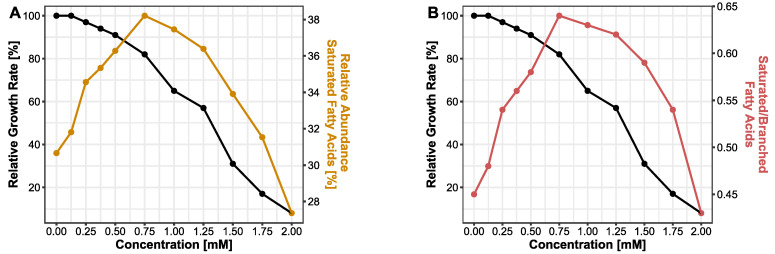
Effect of BPF (0–2 mM) on bacterial membrane lipids. (**A**) Relative abundance of saturated fatty acids. (**B**) Ratio of saturated and branched fatty acids. Both parameters are displayed with the relative growth rate of *B. thetaiotaomicron* during BPF exposure.

It has been demonstrated that bacteria have evolved strategies to cope with their changing habitual conditions in order to survive. These defense mechanisms against chemicals and solvents involve membrane adaptions as well as transport mechanisms. Due to their hydrophobic properties, bisphenols are likely to effect bacterial membranes. Here, we analyzed the changes in the lipid profile of bacterial membranes during bisphenol exposure focusing on BPF. FAME data for BPA and BPS can be found in the supplement (Supplement Figures S3 and S4). As a measure of membrane fluidity, the abundance of saturated fatty acids (14:0, 16:0 and 18:0) was taken into account. In cells treated with a serial increase in BPF concentration, the relative abundance of saturated fatty acids was increasing from 30.7% at 0 mM up to 38.2% at a concentration of 0.75 mM BPF. At this concentration, the curve reached its turning point and the saturation in lipid profile is descending (Figure 2A). The concentration of 0.75 mM BPF corresponds to a relative growth rate of 82%. Additionally, the ratio of saturated fatty acids and branched fatty acids (15:0iso, 15:0anteiso) was observed. Similar to the progression of saturated fatty acids, the ratio of saturated and branched fatty acids is increasing until 0.75 mM and decreasing afterwards (Figure 2B). A decrease in branched fatty acids corresponds to an increase in the SFA/BFA ratio. Hence, an ascending SFA/BFA ratio correlates with a decline in membrane fluidity.

### 3.3. Increase in Acetate Production Indicates Higher Energy Demand Following BPF Exposure

**Figure 3:**
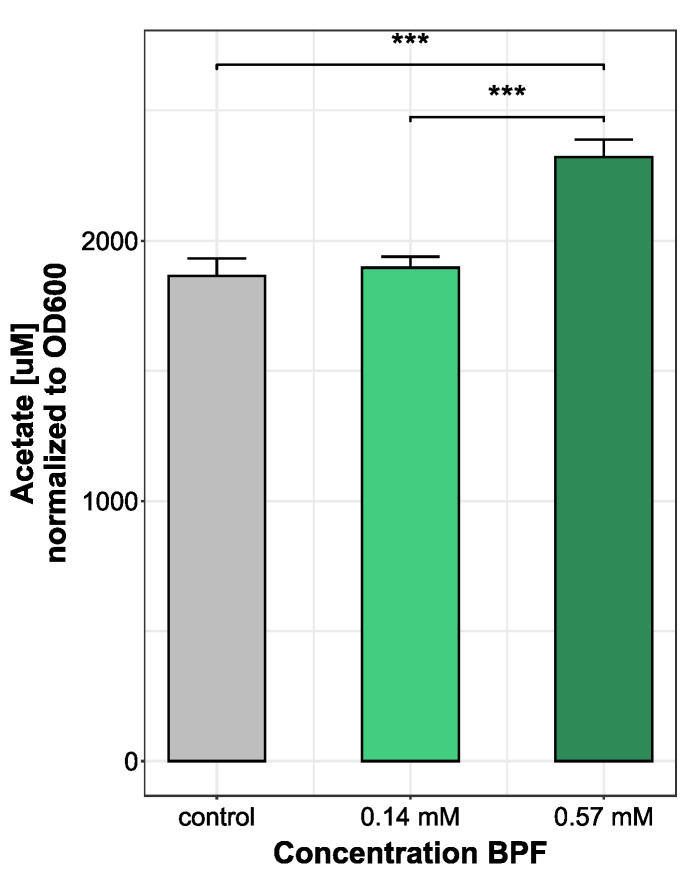
Comparison of acetate production (µM) in *B. thetaiotaomicron* during BPF exposure. *B. thetaiotaomicron* was grown in BHI and exposed to either 50 µg/kg bw (0.14 mM) or 200 µg/kg bw (0.57 mM) BPF. The control contained 0.5% EtOH. Bacteria of all treatments were harvested in late exponential phase and supernatants of cultures were measured using a LC-MS/MS system. SCFA abundance was normalized to OD_600_. Statistical analysis was performed in RStudio running an ANOVA, following a Tukey’s HSD. *** *p*-value < 0.001, *n* = 3.

Observing changes in bacterial metabolism is an approach to gain insight into microbial fitness, especially after an introduced stressor. A change in bacterial metabolite production rate, particularly short chain fatty acids, can be an indirect measure of bacteria–host interaction. Since *Bacteroides thetatiotaomicron* is a bacterium mainly producing acetate, the most abundant SCFA measured was acetate. In total, nine short chain fatty acids were measured. All other short-chain fatty acids were under the limit of detection after medium blank subtraction. Bacteria treated with the highest dose of 0.57 mM BPF showed a significant increase in acetate production (2322.62 ± 66.25 µM) compared to control (1864.3 ± 67.4 µM) and cells grown with 0.14 mM BPF (1896.57 ± 42.21 µM) (Figure 3). During the production of acetate, energy is produced in the form of ATP, which indicates an increased metabolism and higher energy demand during exposure to high concentrations of BPF.

### 3.4. Functional Alterations in the Proteome Show Resistance and Energy Production

**Figure 4:**
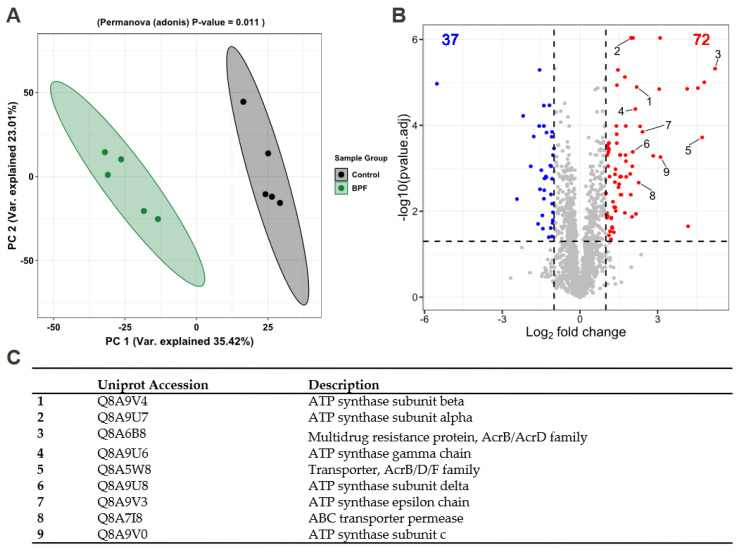
Analysis of the proteome of *B. thetaiotaomicron* exposed to 0.57 mM BPF. (**A**) Principal component analysis based on protein abundances. PERMANOVA *p*-value of 0.01 highlights differences between BPF treatment and control. (**B**) Volcano plot shows differential abundant proteins (students *t*-test, adj. *p*-value < 0.05, Log_2_ fold change > 1). In total, 2050 proteins were detected, among which, 110 were differential abundant in BPF treated cells (decreased: 37 (blue), increased: 72 (red)). Highlighted proteins of interest (1–9) are listed in (**C**).

**Figure 5:**
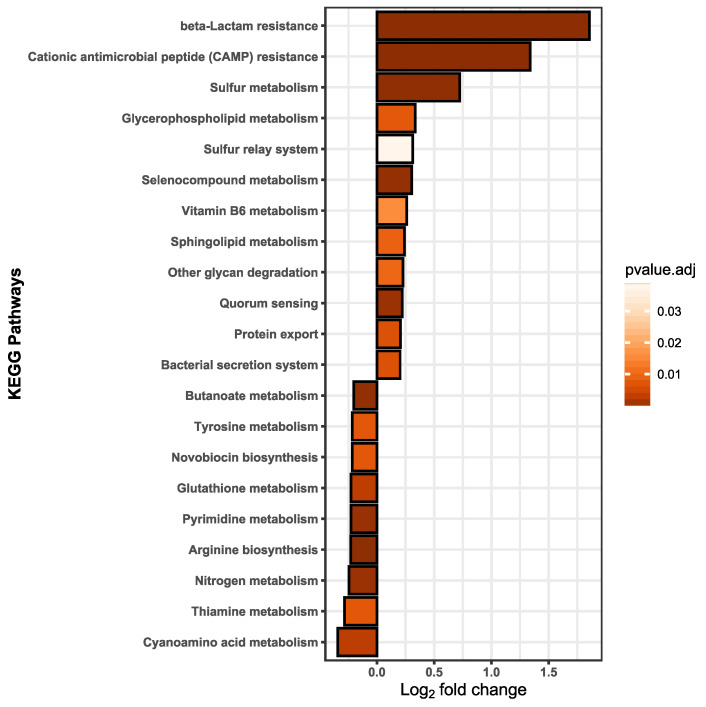
Pathway analysis was based on the summed up protein abundance changes; the pathway function assignments were taken from the KEGG database. Twenty-one pathways were ascertained to be statistically different. A lower fold change indicates a less abundant pathway in BPF treated cells than in control and a fold change in the positive range reflects higher pathway abundance in BPF treated cells than in controls. Statistical analysis was performed in RStudio using multiple t-tests. Results were considered statistically different with a *p*-value adjusted (BH) < 0.05 and an absolute log_2_ fold change > 0.2. *n* = 5.

We assumed that effects on membrane fluidity and increased metabolism are likely linked to functional adaptions that should be detectable on the proteome level. Thus, label-free quantification (LFQ) was performed to detect the relative protein amounts and observe functional changes in *B. thetaiotaomicron* during BPF exposure. Taking into account the relative protein abundances, Figure 4A shows the principal component analysis (PCA) of control and BPF treated cells. Protein identifications are provided (Supplementary Materials Table S7). There is a clear distinction between protein intensities elucidated by a significant permutational multivariate analysis of variance (PERMANOVA, *p*-value: 0.01). Considering changes between control and BPF treated bacteria, log2 fold changes were calculated and differential abundant proteins were quantified. In total, 2050 proteins were detected (4782 proteins in total), from which 110 were considered differential abundant with an adj. *p*-value < 0.05 and a log2 fold change > 1 (37 decreased, 72 increased). Among these proteins, nine were of particular interest, labelled in Figure 4B. These included six ATP-Synthase subunits (Uniprot Accessions: Q8A9V4, Q8A9U8, Q8A9U6, Q8A9U7, Q8A9V0, Q8A9V3), two multidrug resistance family proteins (Uniprot Accession: Q8A6B8, Q8A5W8), and one ABC transporter permease (Uniprote Accession: Q8A7I8) listed in Figure 4C. A list including all differentially abundant proteins is provided (Supplementary Materials Table S4). Additionally, considering changes in the proteome following BPF exposure, 38 and 56 unique proteins for control and treatment were detected. Two proteins solely detected in BPF treated cells were the putative outer membrane protein OprM (Uniprot Accession: Q8A7J4) and an endolytic murein transglycosylase (Uniprot Accession: Q8AAN3), involved in efflux transmembrane transporter activity and murein biogenesis, respectively. A complete list of these proteins from control and treatment can be found in the supplement (Supplementary Materials Table S5). To investigate whether particular functions of *B. thetaiotaomicron* are altered, we summed the LFQ values of proteins assigned to the same pathway from the Kyoto Encyclopedia of Genes and Genomes (KEGG). Among 103 pathways, 21 pathways were considered significantly different in their abundance (adj. *p*-value (BH) < 0.05, absolute log2 fold change > 0.2) between control and BPF treated cells (Figure 5). The two pathways with the highest relative change between control and BPF treatment were beta-lactam resistance and Cationic antimicrobial peptide (CAMP) resistance with log2 fold changes of 1.86 and 1.34, respectively.

## 4. Discussion

Banning BPA in various consumer products and introducing structurally similar substitutes raises the question of analogous adverse effects. Due to the lack of comprehensive microbial effect studies, we present here a study investigating effects of particularly BPF on a commensal human gut bacterium, *B. thetaiotaomicron*. First comparing growth inhibition and membrane lipid profiles after treatment of BPA, BPF, and BPS, we focused on the impact of BPF regarding the SCFA production and functional changes expressed by altered protein abundance and pathway analysis. The concentrations were chosen with the aim to investigate the mode of action of bisphenols in the human microbial gut community and to shed light on the importance of the gut microbiome in respect to xenobiotics exposure.

### 4.1. Impact of Bisphenol A, F, and S on Bacterial Growth

For initial exposure of *B. thetaiotaomicron* to bisphenols, we extrapolated the individual concentration range Figure 1 based on the octanol–water partition coefficient (logP) value of BPA (3.40), BPF (2.91), and BPS (1.63). Compounds whose logP, a value characterizing the hydrophobicity in neutral form [52], is in the positive range (>0) are considered lipophilic [53]. The logP value is a key factor used in drug discovery, environmental fate, and exposure modelling to predict absorption, distribution, and permeation through biological membranes [54,55]. Comparing growth of *B. thetaiotaomicron* during bisphenol exposure, we found that exponential growth was affected most at lower concentrations by bisphenols with ascending logP values. As mentioned in the literature, compounds with a logP > 3 have a relatively high capacity for bioaccumulation in the organism’s tissues [53]. An important characteristic determining the physiological state of the cells is the rate of cell proliferation under steady-state exponential growth, the growth rate [56]. To compare the bacterial proliferation of *B. thetaiotaomicron* exposed to BPA, BPF, and BPS, we determined the relative growth inhibition and inferred the half maximal effective concentration (EC50) of each bisphenol. The EC50 value is common in toxicological studies to evaluate the concentration of, e.g., antibiotics and other pharmaceuticals causing half-maximal response or rather, 50% inhibitory effect on cell growth [57,58]. For the aerobic, BPA-degrading bacterium *Cupriavidus basilensis* JF1, the EC50 was 1.2 mM [59]. In our experiments, a direct correlation between the hydrophobicity (logP) of the tested bisphenols and their toxicity (EC50) was shown (Figure 1B). Depending on their logP values, the bisphenols accumulate in the membranes, affecting their physical properties, especially membrane permeability [60]. However, previous studies found varying growth inhibition by bisphenols in *E. coli* [61]. It has been observed before that each organism has its own intrinsic immunity based on genetics and is influenced by environmental factors [62].

### 4.2. Membrane Adaptions Followed by Bisphenol F Exposure

The cell membrane plays a crucial role in secretion, and transport, the energy status of the cell, signal transduction, and disturbances of the cellular membrane are linked with diminished cell viability [37]. Previous studies showed that non-targeting specific solvents can partition membranes by accumulation and increase fluidity. Thus, permeabilization of the membrane could lead to loss in the electrochemical gradient and finally, cell death [63]. The degree of accumulation of solvents in membranes has been linked with higher toxicity of the compound [37]. This mechanism also has been observed in antibiotics and antimicrobial peptides targeting membranes [64]. Bacteria can cope with environmental stress with various resistance mechanisms. In several bacteria, modification of lipopolysaccharides [65], the degree of saturation of the membrane, or cis/trans isomerization of fatty acids [65] were observed after solvent stress [63,66]. We noticed an increase in saturated fatty acids with ascending BPF concentrations. At concentrations higher than 0.75 mM, the relative abundance of saturated fatty acids declined, a concentration close to the detected EC50 value. An increase in saturated fatty acids means a decline in membrane fluidity, preventing penetration or disruption of the membrane to keep a stable gradient and viable cell [37]. Exceeding the EC50 threshold marks a stressed bacterium, not able anymore to adjust the membrane towards rigidity, since the production of fatty acids can only be achieved by de novo synthesis of membrane lipids, which is a growth- and energy-dependent process [66]. Furthermore, this pattern repeats in the ratio of saturated and branched fatty acids. In Figure 2, a raise in the ratio, until close to the EC50 concentrations, implies a greater number of saturated fatty acids than branched fatty acids in the membrane with ascending BPF concentrations. Increasing the abundance of saturated fatty acids in the cytoplasmic membrane is the major mechanism in the homeoviscous adaption in bacteria, adjusting to the appropriate fluidity of membranes [60,65].

In most cases, Gram-negative bacteria show a higher resistance against, e.g., solvents, due to different membrane characteristics [67]. Being additionally equipped with an outer membrane, an asymmetrical lipid bilayer [68], non-specific chemicals or pharmaceuticals tend to exhibit less disruption than in Gram-positive bacteria [67]. Assuming distinct effects in different bacteria, an important facet will be to monitor the dynamics in a more complex community after bisphenol treatment.

### 4.3. Physiological Changes in Short-Chain Fatty Acid Levels after Bisphenol F Exposure

Short chain fatty acids, the main metabolites from dietary fiber, proteins, and peptides are known immunomodulatory and auxiliary compounds for host health. Particularly, acetate, propionate, and butyrate are the major products in the gut [44]. *B. thetaiotaomicron* is capable of producing the SCFA’s acetate, propionate, and the propionate precursor, succinate [69]. Which SCFAs are formed depends on the type of fermentable substrate, generation time, and incubation period [70]. In media supplemented with readily fermentable carbohydrates (i.e., glucose), the main SCFA produced by *B. thetaiotaomicron* is acetate, whereas propionate is generally favored with complex carbohydrates and under carbon source limitation [71]. After extracting the BHI medium blank, propionate was under the limit of detection in supernatants of cells treated with both BPF concentrations. In some *Bacteroides* species, the main storage product is succinate [72]. Here, only acetate could be detected in concentrations in line with previous studies of *B. thetaiotaomicron* [69,73]. In the main pathway of acetogenesis, pyruvate is decarboxylated, leading to ATP synthesis [74]. Hence, the high concentrations excreted assume acetate as an important energy-conserving by-product of *B. thetaiotaomicron* after BPF exposure, as previously reported for *E. coli* [75]. Acetogenesis has been proposed as a possibility for a more rapid growth to higher cell densities [76] and as a growth advantage against other microorganisms in the presence of carbohydrates [77]. Higher acetate production after BPF treatment indicates a global physical response to fuel adaption mechanisms, as earlier described for *E. coli*, coping with changing proteomic demands of energy biogenesis and biomass synthesis under different degrees of induced stresses [78].

### 4.4. Functional Changes on Proteome Level Induced by Bisphenol F

The need for membrane adaptions after exposure of *B. thetaiotaomicron* to 45 µM BPS in a bacterial community was shown by the altered abundance of two ABC transporters involved in transport of LPS [36]. In our study, we found highly differential abundant proteins associated with the Acriflavine resistance protein family after BPF exposure. These are multidrug resistance efflux pumps, transmembrane transporters that are believed to protect the bacterium against hydrophobic inhibitors (i.e., antibiotics) [79]. AcrB is a known stress protein that can be triggered after solvent, salt, and ethanol stress [80]. It is energized by the proton-motive force and shows a wide substrate specificity. This regulation on the proteome level assumes the penetration and passing through the inner membrane by BPF due to high accumulation potential and pore formation of this rather hydrophobic compound. On the contrary, it has been suggested that toxicity of compounds, especially for gram-negative bacteria, are based on blocking the permeability of the outer membrane, inhibiting the exchange of ions and small molecules [60].

Supporting this hypothesis, we found a significant increase in proteins involved in anaerobic respiration, namely, ATP-synthase subunits in BPF-treated cells. The proton-motive force is required to maintain ATP levels and therefore, fuel the resistance mechanisms against BPF. Wang et al. showed that the maintenance of the proton motive force is essential for expression of antibiotic tolerance in *E. coli*, particularly for Acriflavine resistance proteins [81].

Significantly increased pathways included beta-lactam resistance, cationic antimicrobial peptide (CAMP) resistance, bacterial secretion, and protein export. Altogether, these are defense mechanisms against incoming antibiotics or antimicrobial peptides. Secreting proteins (i.e., proteases) is a first line of defense against AMPs and has been ascertained to commensal human bacteria [82]. Our findings suggest that *B. thetaiotaomicron* can cope with rather high concentrations of BPF; however, this is only achieved through the mobilization of resources (i.e., various resistance mechanisms) and with diminished growth. This could lead to disadvantages with respect to the ability to compete with other species in a gut community and eventually, lead to disturbances and host health effects.

## 5. Conclusions

In this study, we examined the mode of action of bisphenols, in particular, of the BPA substitute BPF, targeting the commensal human gut symbiont *B. thetaiotaomicron*. All tested bisphenols (BPA, BPF, and BPS) showed concentration-dependent growth inhibition, in concentration ranges corresponding to their individual logP values. Changes in membrane fluidity showed that BPF is targeting bacterial membranes. At a BPF concentration of 0.57 mM (200 µg/kg bw), *B. thetaiotaomicron* increased acetate production and exhibited a shift in short-chain fatty acid abundance or composition which could lead to microbiota-directed host health effects. To compensate for growth inhibition or membrane destabilization, *B. thetaiotaomicron* increases the abundance of ATP-synthases, possibly to keep a stable electrochemical gradient. Additionally, on the proteome level, *B. thetaiotaomicron* copes with intruding bisphenols by certain resistance mechanisms, similar to membrane targeting or hydrophobic antibiotics and antimicrobial peptides. These coping mechanisms could diminish the competitive capacity of *B. thetaiotaomicron* in the context of the gut microbiome and eventually lead to disturbance of the homeostatic state of the microbiota. Since each organism (bacterium) has its own intrinsic immunity based on genetics and is influenced by environmental factors [62], the variety of bacteria in the gut has to be taken into account. Future studies should aim toward a more complex bacterial community and illustrate a more realistic approach, including mixtures of chemicals in environmentally relevant concentrations. However, our findings provide an essential concept of the mode of action of BPF. Combined with microbiota-directed effects, this could lead to a better understanding of host health disturbances and disease development based on xenobiotic exposure.

## Data Availability

The mass spectrometry proteomics data have been deposited to the ProteomeXchange Consortium via the PRIDE [83,84] partner repository with the dataset identifier PXD035325.

## Supplementary Materials

The following supporting information can be downloaded at: https://www.mdpi.com/article/10.3390/microorganisms10081610/s1, Figure S1: Linear model growth rate; Figure S2: proteomics median normalization; Figure S3: FAME BPA; Figure S4: FAME BPS; Figure S5: FAME BPF; Table S1: Growth conditions and media; Table S2: Bisphenol concentrations calculations; Table S3: Growth curves and rates; Table S4: Proteomics significant proteins; Table S5: Proteomics unique proteins; Table S6: KEGG pathway analysis; Table S7: Identified proteins.
