# Supplementary material for: Adaptation and Resistance: How Bacteroides thetaiotaomicron Copes with the Bisphenol A Substitute Bisphenol F
Source: Microorganisms. 2022 Aug 9;10(8):1610. doi: 10.3390/microorganisms10081610 (PMC9414779; doi:10.3390/microorganisms10081610)
Supplement: Supplementary file 1 [file microorganisms-10-01610-s001.zip › Supplementary Figure S1_linear model growth rate.pdf]

Supplementary Figure S1 – Diagnostics plots linear models

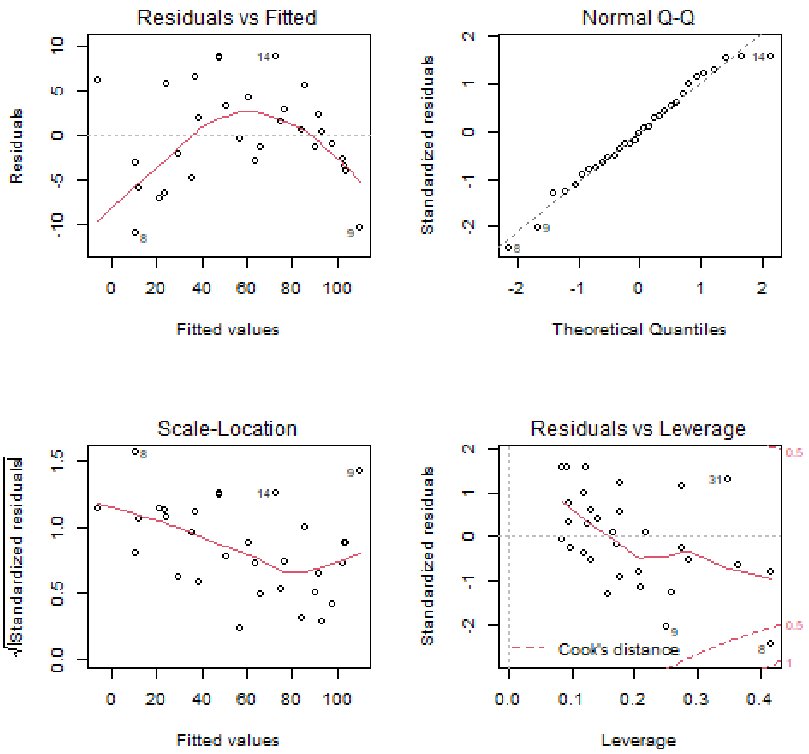


```
Call:
lm(formula = Growth ~ Concentration * Group, data = data)

Residuals:
     Min       1Q   Median       3Q      Max
-11.0000  -3.2817  -0.2905   3.8348   8.9063

Coefficients:
                       Estimate Std. Error t value Pr(>|t|)
(Intercept)            102.6374     3.1425  32.661  < 2e-16 ***
Concentration         -181.3878     9.4660 -19.162  < 2e-16 ***
GroupBPF                 7.6743     4.3053   1.783   0.0868 .
GroupBPS                 0.8626     4.9330   0.175   0.8626
Concentration:GroupBPF 131.7637     9.8481  13.380 6.74e-13 ***
Concentration:GroupBPS 154.9593     9.6390  16.076 1.09e-14 ***
---
Signif. codes:  0 '***' 0.001 '**' 0.01 '*' 0.05 '.' 0.1 ' ' 1

Residual standard error: 5.891 on 25 degrees of freedom
Multiple R-squared:  0.9744,	Adjusted R-squared:  0.9693
F-statistic: 190.5 on 5 and 25 DF,  p-value: < 2.2e-16
```

Figure S1 Diagnostics plot for linear model
