# Supplementary material for: Adaptation and Resistance: How Bacteroides thetaiotaomicron Copes with the Bisphenol A Substitute Bisphenol F
Source: Microorganisms. 2022 Aug 9;10(8):1610. doi: 10.3390/microorganisms10081610 (PMC9414779; doi:10.3390/microorganisms10081610)
Supplement: Supplementary file 1 [file microorganisms-10-01610-s001.zip › Supplementary Figure S2_proteomics_median normalization.pdf]

Supplementary Figure S2 – Boxplot of median normalized protein abundances

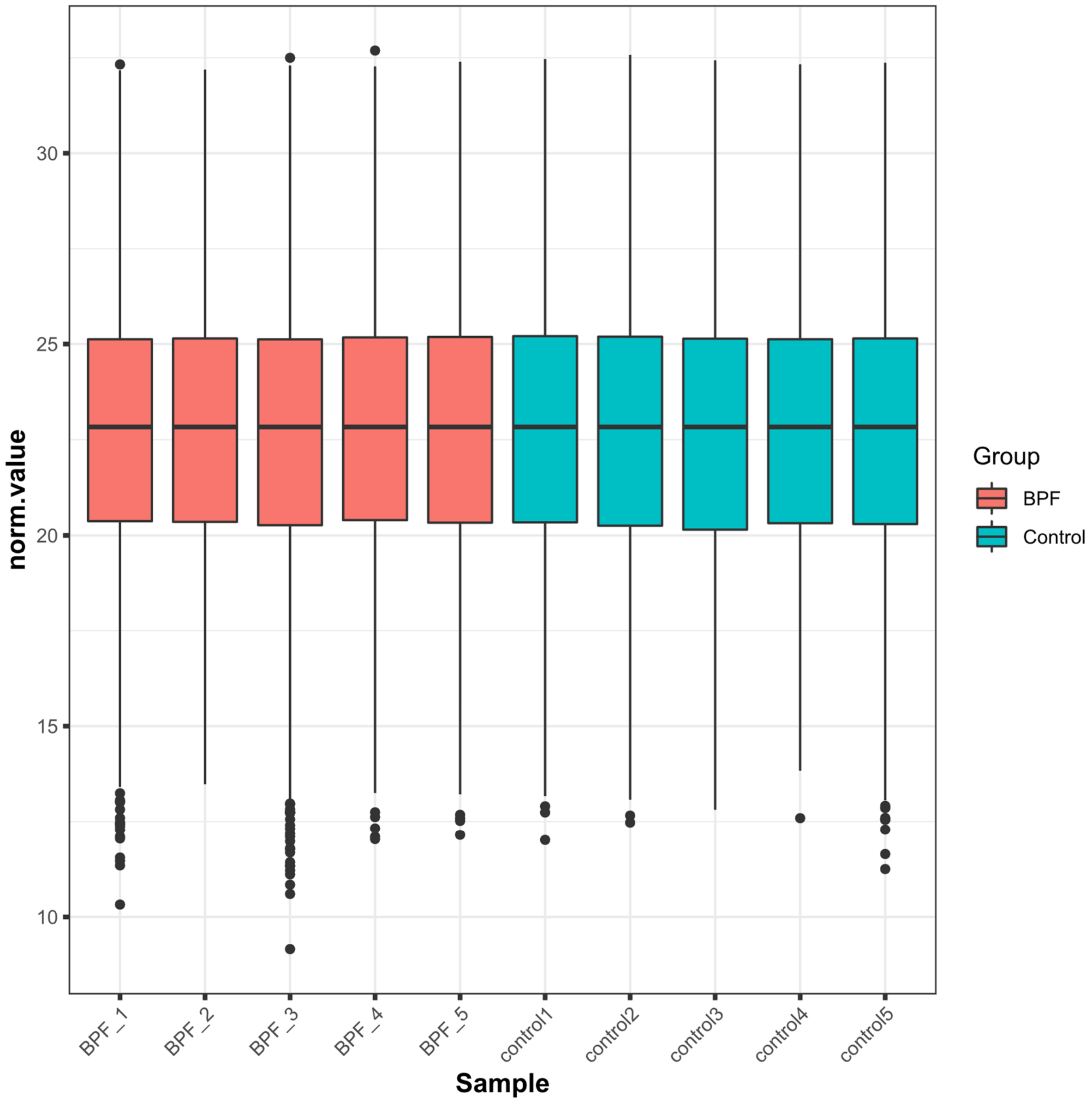

Figure S2 Boxplot of median normalized protein abundances per sample
