# Supplementary material for: Adaptation and Resistance: How Bacteroides thetaiotaomicron Copes with the Bisphenol A Substitute Bisphenol F
Source: Microorganisms. 2022 Aug 9;10(8):1610. doi: 10.3390/microorganisms10081610 (PMC9414779; doi:10.3390/microorganisms10081610)
Supplement: Supplementary file 1 [file microorganisms-10-01610-s001.zip › Supplementary Material Table S1_Growth conditions and media.pdf]

Supplementary Material Table S1: Growth media (Brain-Heart-Infusion, BHI)

Cultivation of *Bacteroides thetaiotaomicron* was performed in complex Brain-Heart-Infusion (BHI) medium with supplements (see Table S1) under anaerobic conditions.

Table S1: Recipe complex Brain-Heart-Infusion (BHI) medium with supplements

Ingredient	Quantity [g or ml/l]	Supplier
Brain-heart-infusion	37	Roth
L-cysteine hydrochloride	0.5	Biochemica
Resazurin	0.001	MP biomedicals
Vitamin k hemin solution	10	Becton Dickinson
Yeast extract	5	Chemsolute

BHI medium was aliquoted into hungates, closed with butyl caps and immediately purged with nitrogen. Autoclaved at 121°C for 20 min and stored at 4°C until use. For growth experiments hungates were inoculated to an OD_{600nm} of ~0.1 with freshly thawed bacterial cells. Bacteria were incubated at 37°C and 175 rpm.
