# Supplementary material for: Adaptation and Resistance: How Bacteroides thetaiotaomicron Copes with the Bisphenol A Substitute Bisphenol F
Source: Microorganisms. 2022 Aug 9;10(8):1610. doi: 10.3390/microorganisms10081610 (PMC9414779; doi:10.3390/microorganisms10081610)
Supplement: Supplementary file 1 [file microorganisms-10-01610-s001.zip › Supplementary Material Table S2_Concentration calculations bisphenols.pdf]

Supplementary Material Table S2: Concentration calculations bisphenols

Table S2 Characteristics of used bisphenols

Chemical	M [g/mol]	Solvent
Bisphenol A	228.29	Ethanol
Bisphenol F	200.24	Ethanol
Bisphenol S	250.27	Ethanol

Exposure concentrations for metabolomic and proteomic analysis of bisphenol F were calculated according to a 70 kg person with a daily intake of 50 µg/kg or 200 µg/kg. The average stool weight per day [1] was included in the calculations.

50 µg/kg body weight:

$$\frac{(50\ \mu g/kg * 70\ kg)}{123.6\ g} = 28.32\ \mu g/g$$

200 µg/kg body weight:

$$\frac{(200\ \mu g/kg * 70\ kg)}{123.6\ g} = 113.27\ \mu g/g$$

We converted µg/g of stool into mg/ml, due to our liquid cultivation in batch cultures. This lead to 0.0283 mg/ml and 0.113 mg/ml for 50 µg/kg and 200 µg/kg, respectively. Molar concentrations (mM/L) were calculated for each bisphenol according to their specific Molar mass. For bisphenol F this corresponds to 0.14 mM/L and 0.57 mM/L for 50 µg/kg and 200 µg/kg, respectively.

1. Rendtorff, R.C.; Kashgarian, M. Stool patterns of healthy adult males. *Dis Colon Rectum* **1967**, *10*, 222-228, doi:10.1007/BF02617184.
