# Supplementary figures and images for: Adaptation and Resistance: How Bacteroides thetaiotaomicron Copes with the Bisphenol A Substitute Bisphenol F
Source: Microorganisms. 2022 Aug 9;10(8):1610. doi: 10.3390/microorganisms10081610 (PMC9414779; doi:10.3390/microorganisms10081610)

Supplementary Figure S3 – FAME BPA

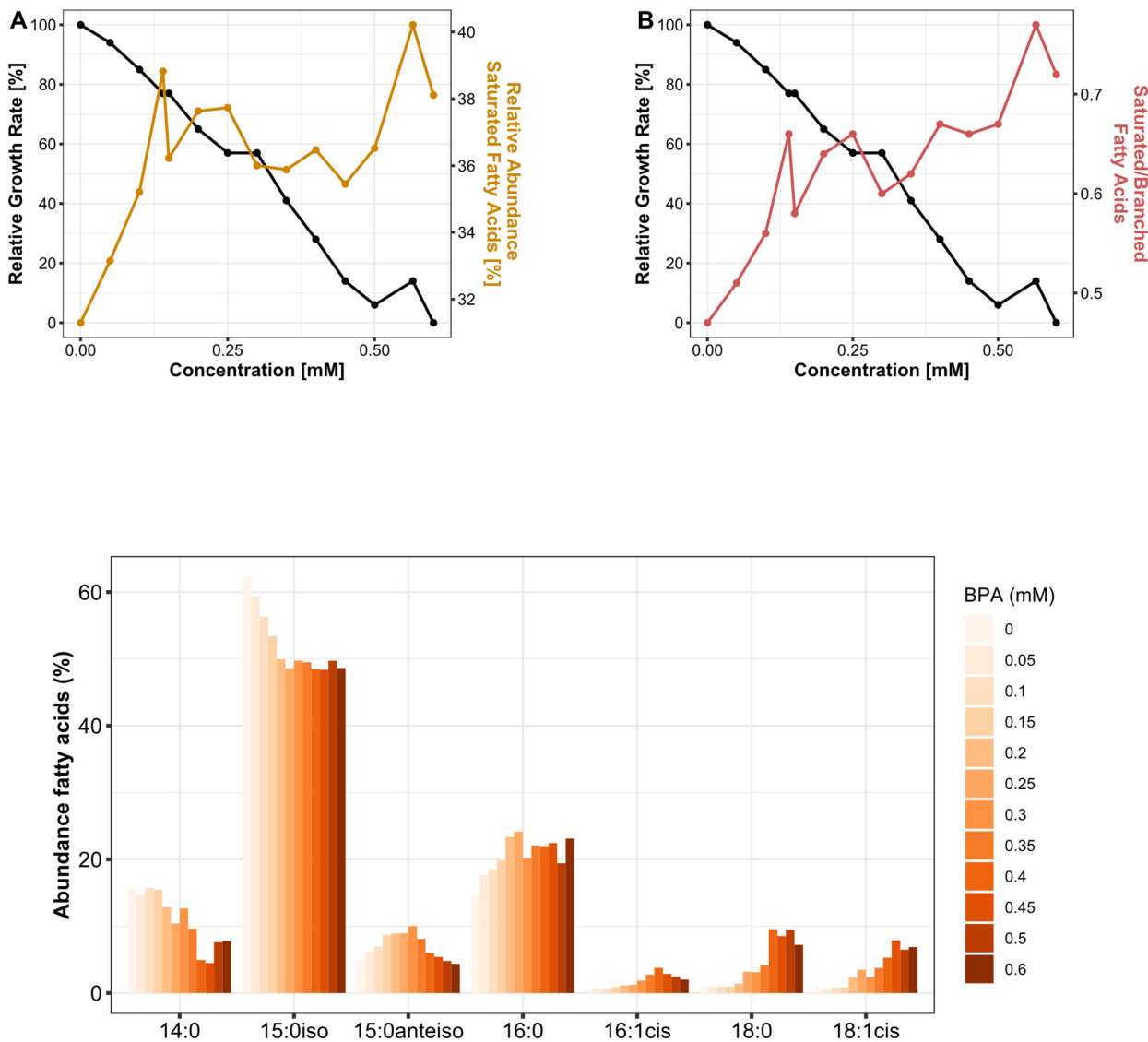

Supplement: Supplementary file 1 [file microorganisms-10-01610-s001.zip › Supplementary Figure S3_FAME BPA.pdf]

Supplementary Figure S4 – FAME BPS

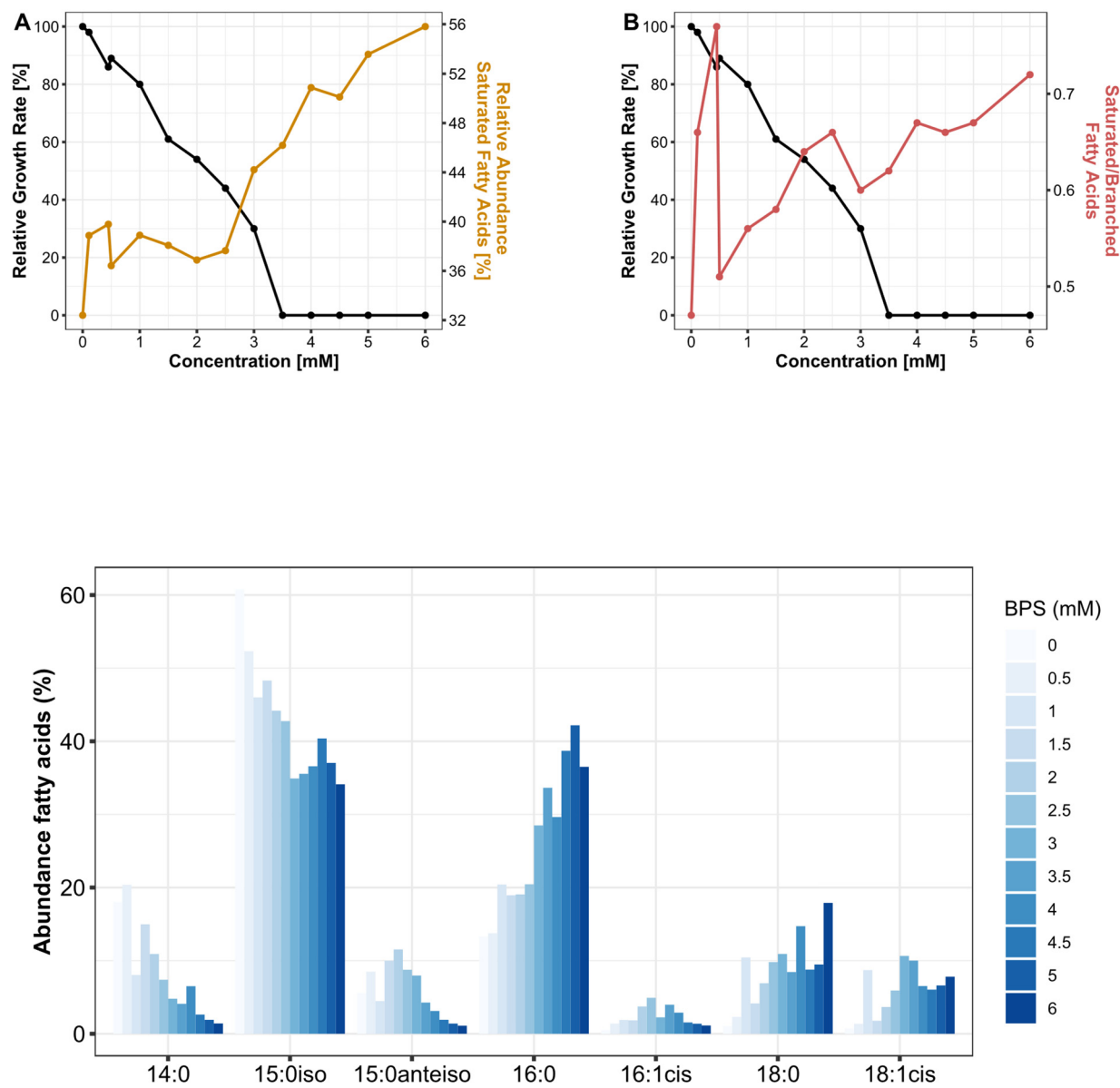

Supplement: Supplementary file 1 [file microorganisms-10-01610-s001.zip › Supplementary Figure S4_FAME BPS.pdf]

Supplementary Figure S5 – FAME BPF

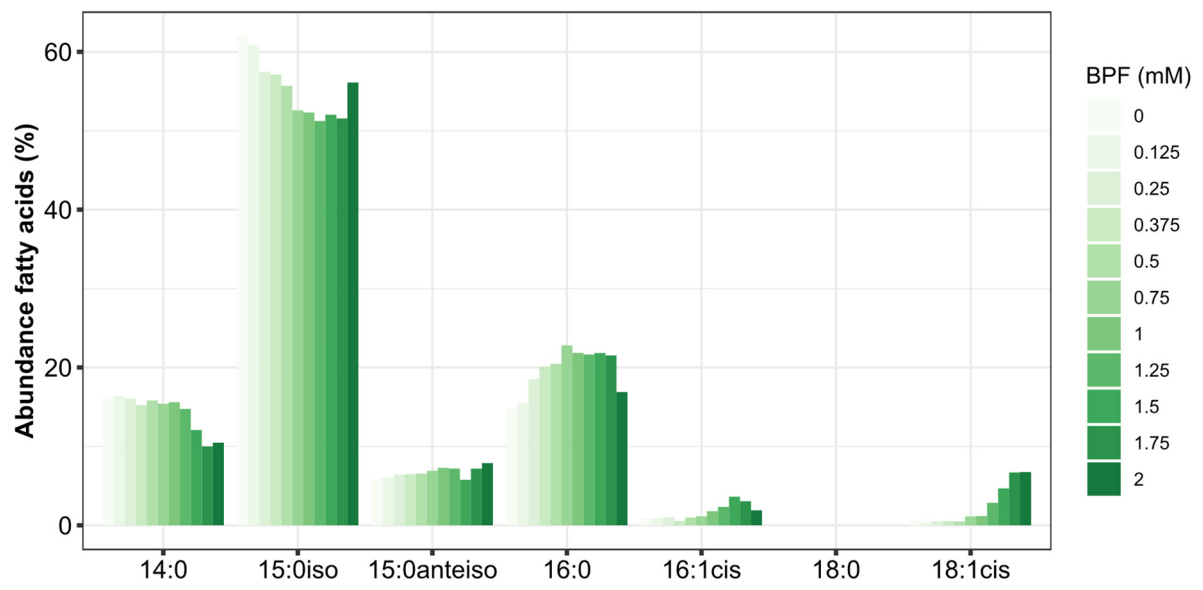

Supplement: Supplementary file 1 [file microorganisms-10-01610-s001.zip › Supplementary Figure S5_FAME BPF.pdf]
